# Transcriptional Reactivation of the *FMR1* Gene. A Possible Approach to the Treatment of the Fragile X Syndrome†

**DOI:** 10.3390/genes7080049

**Published:** 2016-08-17

**Authors:** Elisabetta Tabolacci, Federica Palumbo, Veronica Nobile, Giovanni Neri

**Affiliations:** Institute of Genomic Medicine, School of Medicine, Catholic University, Largo Francesco Vito 1, Rome 00168, Italy; elisabetta.tabolacci@unicatt.it (E.T.); palumbo.federica@tiscali.it (F.P.); veronicanobile88@gmail.com (V.N.)

**Keywords:** Fragile X syndrome, *FMR1* gene, epigenetic therapy, DNA methylation, histone modifications, drug treatments

## Abstract

Fragile X syndrome (FXS) is the most common cause of inherited intellectual disability, caused by CGG expansion over 200 repeats (full mutation, FM) at the 5′ untranslated region (UTR) of the fragile X mental retardation 1 (*FMR1*) gene and subsequent DNA methylation of the promoter region, accompanied by additional epigenetic histone modifications that result in a block of transcription and absence of the fragile X mental retardation protein (FMRP). The lack of FMRP, involved in multiple aspects of mRNA metabolism in the brain, is thought to be the direct cause of the FXS phenotype. Restoration of *FMR1* transcription and FMRP production can be obtained in vitro by treating FXS lymphoblastoid cell lines with the demethylating agent 5-azadeoxycytidine, demonstrating that DNA methylation is key to *FMR1* inactivation. This concept is strengthened by the existence of rare male carriers of a FM, who are unable to methylate the *FMR1* promoter. These individuals produce limited amounts of FMRP and are of normal intelligence. Their inability to methylate the *FMR1* promoter, whose cause is not yet fully elucidated, rescues them from manifesting the FXS. These observations demonstrate that a therapeutic approach to FXS based on the pharmacological reactivation of the *FMR1* gene is conceptually tenable and worthy of being further pursued.

## 1. Introduction

Hereditary disorders of the epigenetic machinery are a newly delineated group of multiple congenital anomalies and intellectual disability (ID) syndromes resulting from mutations in genes encoding components of the epigenetic machinery (acting *in trans*) or epigenetic alterations at specific loci (acting *in cis*), such as Rett syndrome and Kleefstra syndrome. A paradigmatic condition belonging to this latter group is the Fragile X syndrome (FXS, OMIM #300624), an X-linked condition with hemizygous males more severely affected than females, characterized by cognitive impairment, behavioral abnormalities (i.e., anxiety, attention deficit with hyperactivity disorder, social shyness), muscular hypotonia and some physical signs (i.e., long face, large ears, prominent jaw, macroorchidism) [1]. Up to 67% of FXS boys meet either the autism disorder (AD) or autism spectrum disorder (ASD) criteria [2]. In affected individuals examined post-mortem, a higher density of dendritic spines was found, suggesting a possible failure of synapse elimination. While variously misshapen spines are characteristic of a number of ID syndromes, the overabundance of spines seen in FXS is unusual [3]. A recently meta-analysis sets the frequency of affected males at 1.4:10,000 and that of affected females at 0.9:10,000, while the prevalence of female carriers of the FXS premutation in the normal population is 1:291, according to one study [4]. The name FXS was derived from the folate-sensitive fragile site FRAXA (fragile site, X chromosome, A site) on the long arm of chromosome X (Xq27.3-Xq28) in affected males [5], corresponding to the locus of the causative gene of the syndrome, *FMR1*, which contains in its 5′ untranslated region (UTR) a polymorphic CGG triplet repeat [6]. FXS is almost exclusively caused by expansion of the CGG triplet over 200 repeats (full mutation, FM), followed by cytosine methylation of both the CGGs and CpG island located in the upstream promoter region (methylated full mutation, MFM). This epigenetic change is accompanied by histone modifications typical of a heterochromatic status, incompatible with transcription, thus preventing translation of the FMRP protein. The causative FXS mutation is a typical loss-of-function mutation, caused by both a sequence anomaly (CGG expansion) and epigenetic modifications (DNA methylation and histone changes), in the presence of an intact coding sequence of the gene. The absence of FMRP, an RNA-binding protein mainly involved in several aspects of mRNA metabolism particularly in the brain and in synaptic formation and maturation (for a review see [7]), is the ultimate cause of the FXS phenotype.

A number of CGG between 55 and 200 defines another class of alleles, the premutated alleles (PM). The PM alleles are at high risk of expanding into FM alleles through maternal meiosis and confer a risk to develop fragile X-associated tremor/ataxia syndrome (FXTAS, OMIM #300623) and fragile X-associated primary ovarian insufficiency (FXPOI, OMIM #300624). Overall, these disorders, known as FRAXopathies [8] or fragile X-related disorders [9], share the CGG triplet instability of the *FMR1* gene but are characterized by opposed epigenetic changes, with decreased transcription in FXS patients (loss-of-function) and increased transcription in PM carriers, resulting in a toxic gain-of-function of the excess mRNA [10].

This scenario is complicated by the description of rare individuals with apparent normal phenotype, carriers of an unmethylated full mutation (UFM) with a CGG triplet over 200 repeats, which is completely unmethylated and retains an euchromatic configuration, compatible with both transcription and translation of the gene, even though at higher and lower levels respectively, compared to normal [11,12,13]. In a chorionic villus sample (CVS) of a FXS fetus the absence of FMRP was observed at around 11 weeks of gestation due to hypermethylation of the expanded CGG sequence [14], but the mechanisms through which a FM becomes methylated in FXS patients and not in UFM individuals remain unclear. In a recent paper it was shown that *FMR1* silencing in human embryonic stem cells (hESCs)-derived neurons is mediated by its own mRNA which hybridizes to the complementary CGG repeat on the DNA sequence to form an RNA:DNA duplex. Disrupting the interaction of the mRNA with the CGG repeat seems to prevent silencing of the promoter, supporting a mechanism of RNA-directed gene silencing. However, it should be noted that the hESCs employed in this work were already partially methylated, casting some doubts on the conclusions reached by the authors [15]. In any case, this mechanism does not explain the existence of UFM individuals, who preserve the *FMR1* transcription in presence of CGG expansion. It should be noted that *FMR1* epigenetic gene silencing takes place already in FXS-hESCs. Using 12 FXS-hESCs it was observed that *FMR1* hypermethylation occurs during the undifferentiated state and thus earlier than 11 weeks and it is tightly linked to *FMR1* transcriptional inactivation [16].

### 1.1. The FMR1 Gene and Its Protein Product

The *FMR1* gene was identified by positional cloning by Verkerk et al. [6]. It contains 17 exons spanning 38 kb [17] and produces a 4.4 kb mRNA, which results in the generation of 12 different isoforms of FMRP protein through alternative splicing [18], with a molecular weight of 70–80 kDa.

The *FMR1* locus includes (from 5′ to 3′) an upstream methylated region, the promoter region, the CGG stretch and the coding sequence. The boundary of the methylated region is located approximately 650–800 nucleotides upstream from the CGG sequence. In FXS alleles this boundary is no longer visible, given that promoter and CGG stretch are also methylated, while it is preserved in UFM alleles [19,20]. The methylation boundary region contains binding sites for various nuclear proteins, including CTCF (CCCTC-Binding Factor), a possible transcriptional regulator for this locus. This binding is likely to prevent methylation spreading towards the *FMR1* promoter region. CTCF protein was recently considered as a possible regulator of *FMR1* expression, probably modulating its transcription through chromatin loop formation [20].

The promoter contains approximately 56 CpG sites (CpG island) and includes three initiator-like (InR) sequences localized about 130 nucleotides (nt) upstream the CGG sequence. The transcription starts from one of these three transcription start sites within a region of approximately 50 nt, and the size of the CGG repeat may act as a downstream enhancer/modulator of transcription. Initiation shifts to the upstream sites when the size of the CGGs expands [21].

**Figure 1:**
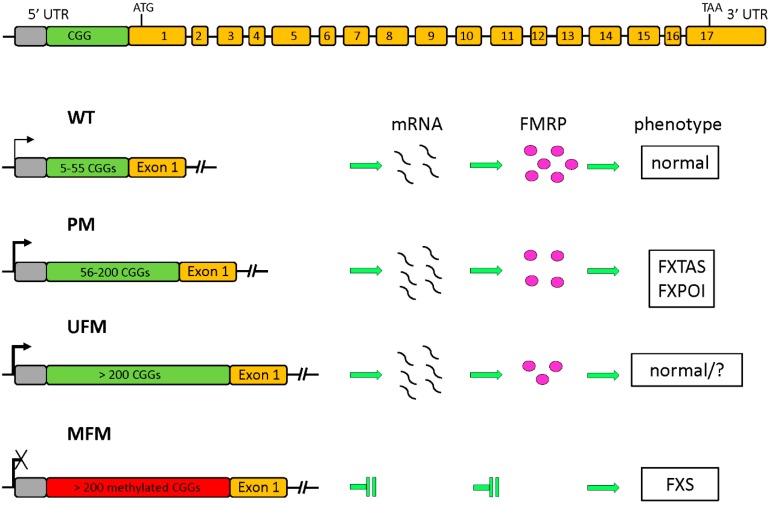
Structure of the fragile X mental retardation 1 (*FMR1*) gene (top) and its various allelic forms. The polymorphic CGG sequence is placed upstream of exon 1 in the 5′ untranslated region (UTR). Based on the CGG expansion four different classes of alleles are shown, with their transcriptional activity indicated by the arrow: normal (WT); premutated (PM) with a higher transcription (bold arrow) and slight decrease of translation associated to the fragile X-associated tremor/ataxia syndrome (FXTAS) end fragile X-associated primary ovarian insufficiency (FXPOI) phenotypes; unmethylated full mutation (UFM), similar to PM for transcription and translation, without an apparent phenotype; methylated full mutation (MFM) leading to absence of transcript and fragile X mental retardation protein (FMRP) and consequently to fragile X syndrome (FXS).

The polymorphic CGG repeat is located in the 5′ UTR of exon 1. Based on the CGG expansion range, three main classes of alleles are described: normal, with 5–55 CGGs; PM, with 56–200 CGGs and FM, with over 200 CGGs. These latter alleles are subject to hypermethylation and epigenetic silencing, with consequent loss of FMRP. This loss-of-function mutation is the cause of the FXS phenotype. A schematic overview of the *FMR1* gene structure with the main classes of alleles and their transcriptional activity is depicted in Figure 1. These three ranges of expansion correlate with a different transmission pattern through generations: normal alleles are stable; above 56 repeats the alleles become progressively unstable and can expand to FM during maternal meiosis. So far, the smallest allele capable of switching from PM to FM in a single maternal meiosis was found to contain 56 CGGs [22]. No case has yet been described of direct expansion from normal to FM. One or two AGG triplets may be interspersed every 9–10 CGGs [17]. The presence of these AGGs keeps the CGGs stable during DNA replication, and their presence lowers the risk for maternal PM to be passed down as a FM [23]. It has been reported that the presence of two interspersed AGGs leads to a 60% decrease of such risk, in women carrying 70–80 CGGs [24]. Because the number of triplets expands over the generations, the number of affected individuals increases accordingly. This phenomenon was called the Sherman paradox [25].

The protein product of *FMR1*, FMRP can be primarily classified as an RNA-binding protein that regulates translation of several target mRNAs, and particularly those associated with neuronal development. The interaction with target mRNAs is mediated by two K homology (KH) domains and an arginine-glycine-glycine triplet (RGG) box in the central region and C-terminus of the protein, respectively. The protein has a nuclear localization signal (NLS) and a nuclear export signal (NES), used to shuttle from nucleus to cytoplasm and back. The fraction of the protein that stays within the nucleus is only about 4%, the balance being localized in the cytoplasm [26]. FMRP can form homodimers and interacts with several cytoplasmic and nuclear proteins involved in mRNA metabolism, including RNA interference (RNAi) [27,28] and RNA editing [29]. It also has a role in cytoskeleton remodeling, via its N-terminal and central regions. FMRP interactions and functions may be modulated by its post-translational modifications, such as phosphorylation at amino acids 483 and 521 [30].

**Figure 2:**
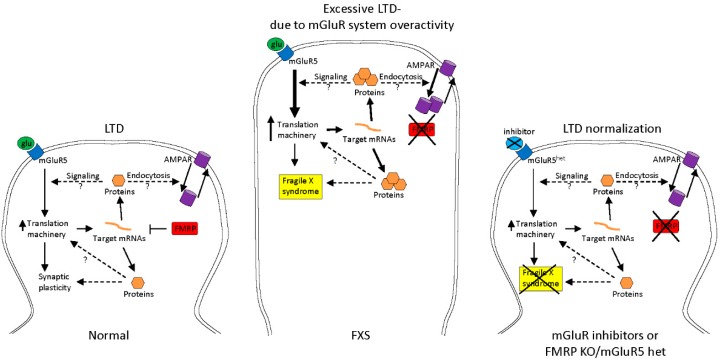
Schematic representation of the metabotropic glutamate receptor (mGluR) theory. In normal dendrites FMRP inhibits translation of pre-existing mRNAs after mGluR stimulation, with normal ionotropic glutamatergic receptor (AMPAR) endocytosis. FXS dendrite appears thinner compared to normal, because mGluR stimulation by glutamate causes an excess of local protein synthesis with exaggerated long term depression (LTD) due to a higher AMPAR endocytosis activity. The use of a mGluR inhibitor leads to a normalization of LTD in absence of FMRP.

There is evidence that the absence of FMRP results in an increased translation rate of its target mRNAs [31], which is consistent with the idea that FMRP functions as a repressor of translation, especially at synapses. An example of this mechanism is given by the excess of protein synthesis secondary to the lack of FMRP, observed after metabotropic glutamate receptor type 5 (mGluR5) stimulation of *Fmr1* knock-out mice synapses and represents the basis for the so-called “mGluR theory” [32]. The absence of FMRP might also induce an increase in the translation of proteins involved in internalization of α-amino-3-hydroxy-5-methyl-4-isoxazolepropionic acid (AMPA) receptors (ionotropic glutamate receptors), which could lead to elongation of the dendritic spine and fewer glutamate ionotropic receptors on the post-synaptic membrane (Figure 2). *Fmr1* knockout mice, heterozygous for mGlur5 (50% reduction of expression of this receptor) showed a rescue of the synaptic phenotype [33].

In addition to its role in the brain, FMRP plays important functions in other cell types, such as tumor cells. Its mRNA is overexpressed in hepatocarcinoma cells [34,35] and FXS patients seem to have lower risk of developing cancer [36,37]. Recently, it was shown that high levels of *FMR1*-mRNA in human breast cancer cells increase the probability for these cells to form lung metastases [38].

Within the nucleus, FMRP acts as a chromatin-binding protein, with a function in the DNA damage response (DDR), playing an important role in gametogenesis. It has been shown that FMRP occupies meiotic chromosomes and regulates the dynamics of the DDR machinery during mouse spermatogenesis [39].

### 1.2. Epigenetic Regulation of FMR1 Transcription

The silencing of the *FMR1* gene seems to require several levels of epigenetic regulation (DNA methylation, histone modifications, chromatin remodeling and RNAi), although the critical mechanism involved in the silencing is not fully understood [40]. Silenced FM alleles have a heterochromatic configuration, transcriptionally non permissive, compared to transcriptionally active wild type (WT) alleles, which are characterized by an “open” euchromatic configuration, permissive for transcription. Generally, switching from active transcription to transcriptional silencing is a direct consequence of CGG repeat expansion over 200 units and its consequent epigenetic modifications. An exception to this rule is represented by the UFM alleles, in which *FMR1* transcription persists despite CGG expansion over 200 repeats.

In FM alleles, the cytosines of the CpG island and of the expanded CGGs become methylated, histones 3 and 4 (H3 and H4) are deacetylated, lysine 4 on H3 (H3K4) is demethylated, while lysine 9 on H3 (H3K9) becomes methylated, lysine 27 on H3 is trimethylated (H3K27me3) and lysine 20 on H4 (H4K20) increases its methylation status near the CGG expansion [41,42,43]. All these epigenetic marks define a heterochromatin configuration, transcriptionally non permissive.

In contrast to the hypoacetylation of FM alleles, PM alleles have 1.5–2 times the normal levels of acetylated H3 and H4 [44], thought to be responsible for their increased transcription [45]. These epigenetic modifications confer a more open chromatin structure to the *FMR1* promoter and RNAs transcribed from premutated CGG expansions tend to form hairpins, which may account for the stalling of the 40S ribosomal subunits and consequently for the translation deficit of PM alleles [46]. The increased transcription of PM alleles may represent a mechanism to compensate for the lower rate of FMRP translation [47,48]. The excess of *FMR1*-mRNA was detected in the inclusions of both neurons and astrocytes of FXTAS patients [49]. This pathogenic model is typically gain-of-function. Although the pathogenesis of FXPOI, the second PM-associated phenotype, is still unknown, it seems to be associated with a similar toxic gain-of-function mechanism. This landscape is further complicated by the existence of UFM alleles, identified in individuals with apparently normal intelligence, belonging to FXS families [10,11,12]. Their *FMR1* is transcriptionally active, despite a CGG expansion above 200 repeats. The DNA is unmethylated and the histone marks are similar to those of WT alleles (H3 and H4 acetylated, H3K4 methylated and H3K27 dimethylated), with the exception of H3K9 which remains partially methylated like in FM alleles. The major epigenetic modifications in normal, FXS and UFM alleles are reported in Table 1. UFM alleles represent the status of FXS cells before FMs are silenced at around 11 weeks of gestation [14]. Studies in human ESCs showed that DNA methylation is the last step before silencing of the *FMR1* gene occurs. In FX-ESCs, H3K9 dimethylation of the *FMR1* promoter was detected before the occurrence of DNA methylation [50] and in induced Pluripotent Stem (iPS) cells derived from FXS fibroblasts the reprogramming did not influence the DNA methylation and the histone modification at the *FMR1* locus, which remained in a heterochromatic configuration [51]. In FXS-hESCs *FMR1* hypermethylation with its consequent transcriptional inactivation occurs during the undifferentiated state and it is associated to loss of H3K4me2, gain of H3K9me3 and is unrelated to CTCF binding [16].

To identify structure-specific proteins that could recruit components of the silencing machinery, we explored the role of two proteins. The first was the DNA binding protein CTCF, which was recently considered as a possible regulator of *FMR1* transcription [20]. CTCF binding is absent in methylated FM alleles and present in UFM alleles. Notably, pharmacological demethylation with 5-aza-2-deoxycytidine (5-azadC) of FXS cells did not restore CTCF binding to the *FMR1* gene. CTCF depletion with siRNA caused a reduction of both *FMR1* and FMR1 antisense RNA 1 (*FMR1-AS1*) transcription, which however did not appear to be caused by re-methylation of the *FMR1* promoter, both in normal and UFM cell lines. Based on these findings, we concluded that CTCF may have a complex role in regulating *FMR1* expression, probably through the organization of chromatin loops between sense/antisense transcriptional regulatory regions. The second protein investigated was the CGG-binding protein (CGGBP1) [52]. CGGBP1 is a highly conserved protein that binds specifically to unmethylated CGG tracts. Chromatin immunoprecipitation (ChIP) assays clearly demonstrated that CGGBP1 binds to unmethylated CGG triplets of the *FMR1* gene, but not to methylated CGGs. However, CGGBP1 silencing with shRNAs did not affect *FMR1* transcription and CGG expansion stability in expanded alleles. Although the strong binding to the CGG tract could suggest a role of CGGBP1 on *FMR1* gene expression, these data demonstrate that CGGBP1 has no direct effect on *FMR1* transcription and CGG repeat stability.

Transcription of the *FMR1* locus includes several noncoding RNAs (ncRNAs), particularly long ncRNAs (lncRNAs), which can be transcribed from both strands of the gene, sense and antisense. These lncRNAs may act as modulators of transcription or of the epigenetic landscape of the locus of origin. The main ncRNA originated from the *FMR1* locus is *FMR1-AS1*, an antisense transcript absent in FM alleles and upregulated in PM and UFM alleles [20,53]. It is alternatively spliced, polyadenilated and exported to the cytoplasm. It appears to be driven by two alternative promoters: one is the *FMR1* bidirectional promoter and the second is located in the second intron of the *FMR1* gene. In both cases it includes the CGG∙GGC repeat sequence. PM and UFM alleles showed a specific alternative splicing in intron 2 that used a non-consensus CT-AC splice site. Other ncRNAs were further identified, both in the sense and in the antisense direction. *FMR4* is transcribed in the antisense orientation, is absent in the FM alleles and slightly overexpressed in PM alleles [54]. Additional ncRNAs were recently identified: *FMR5* and *FMR6* [55]. The former is a sense lncRNA transcribed from 1 kb upstream of the *FMR1* transcription start site (in the methylated region upstream of the methylation boundary) and is expressed in WT, PM and FM alleles. The latter is an antisense transcript that overlaps exons 15, 16 and 17, as well as the 3′ UTR, splicing out the introns through non-canonical consensus sites, and is silenced in expanded alleles (both PM and FM). Although the exact role of these ncRNAs is not yet elucidated, they could act as regulators of *FMR1* transcription, possibly acting as scaffold for the proteins necessary for heterochromatin formation or as a guide for the recruitment of silencing complexes, as described for other loci through RNAi [56]. However, knockdown of Dicer, Argonaute 1 (Ago1) and Argonaute 2 (Ago2), which play a key role in RNAi, did not prevent *FMR1* gene silencing in FXS-hESC derived-neurons, ruling out the involvement of RNAi in silencing FM alleles [15]. At the same time, FMR1 mRNA has been proven capable of forming RNA:DNA hybrids (R-loops) during transcription when the CGG tract reaches at least the premutation size [57]. These structures are commonly formed at expanded repeat loci by the persistent pairing of the nascent mRNA with the DNA template strand, leaving the non-template DNA strand unpaired. Furthermore, R-loop formation may be facilitated by hairpin formation on the non-template (CGG-containing) strand that would reduce the likelihood of reannealing of the two DNA strands [58]. It has also been proven that even more stable R-loops are formed when UFM alleles are transcribed, as in FXS-hESC [15] and after pharmacological *FMR1* reactivation in a FXS lymphoblastoid cell line [59]. The different stability of the R-loop (transient in PM and more prolonged in FM) and the probably different conformation of the unpaired CGG-containing sense strand (more linearized in PM, probably rich in hairpins in FM) results in opposite results: the more stable R-loop with longer FM alleles blocks transcription initiation (and elongation) eventually silencing FM [15,59] while the unpaired non-template strand of PM would actually recruit transcription activators [59]. Therefore, repeat-induced R-loop formation would have opposing effects depending on its total length: PM alleles would result in more active local chromatin with increased *FMR1* transcription while longer FM alleles would rather block transcription and effectively induce local heterochromatin formation [58].

## 2. Therapeutic Approaches for FXS

Two main mechanisms are involved in *FMR1* gene silencing: CGG expansion over 200 repeats and epigenetic modifications (mainly DNA methylation), in presence of an intact open reading frame (ORF). These mechanisms lead to the absence of FMRP, whose lack causes the FXS phenotype. Thus, two different approaches could in principle be employed to treat FXS: (a) to normalize the defective functions due to the lack of FMRP, acting on the pathways in which it is involved; and (b) to restore *FMR1* expression acting on the epigenetic mechanisms involved in the transcriptional inactivation. Both approaches were tested in vitro (mainly on *Fmr1* knock-out mouse brain slices and FXS patient cells) and in vivo (in animal models and in clinical trials). All clinical trials so far were based on evidence obtained on animal models, both mouse and *Drosophila*. It would be of the utmost importance to test new therapeutic approaches on a human cellular model. FXS-iPS cells were obtained by different groups, and neurons derived from FXS-iPS cells represent a potentially useful cellular model to test new drugs. 

### 2.1. Treatments to Compensate for Lack of FMRP

According to plan (a) above, several clinical trials were conducted, all aimed at correcting the FXS synaptic defect. Most of them stemmed from the discovery of excessive mGLuR signaling at synapses lacking FMRP [32]. Interesting results were obtained with AFQ056 (Novartis), a selective inhibitor of mGluR5, on 30 FXS males aged 18–35 years, showing a significant amelioration of hyperactivity in a subgroup of drug-treated patients [60]. This subgroup had a complete methylation of the *FMR1* promoter, while in the non-responder subgroup DNA methylation was incomplete. The hypothesis that AFQ056 may affect DNA methylation was not supported by an in vitro study [61]. Unfortunately, these encouraging preliminary results were not confirmed by subsequent trials with AFQ056. Several other pharmacological trials were performed to try and compensate for the altered function of specific neuronal receptors or pathways, consequent to lack of FMRP (reviewed in [62]). Considering the large number of mRNAs targeted by FMRP and the various dysregulated pathways, most of these clinical trials with a single drug were inconclusive, probably due to compensation mechanisms within a very complex scenario (mRNAs, pathways and/or FXS clinical phenotype) [63].

### 2.2. Epigenetic Treatments

The second approach (plan (b) above) to treat FXS is based on the possibility to revert the epigenetic marks which maintain the mutated *FMR1* gene silent. The hypothesis of reversibility is supported by the existence of UFM individuals who for some yet unknown reasons are unable to methylate their fully expanded CGG tract allowing *FMR1* transcription. This approach may be theoretically considered more effective in curing FXS because it goes directly to the cause of the transcriptional silencing.

**Figure 3:**
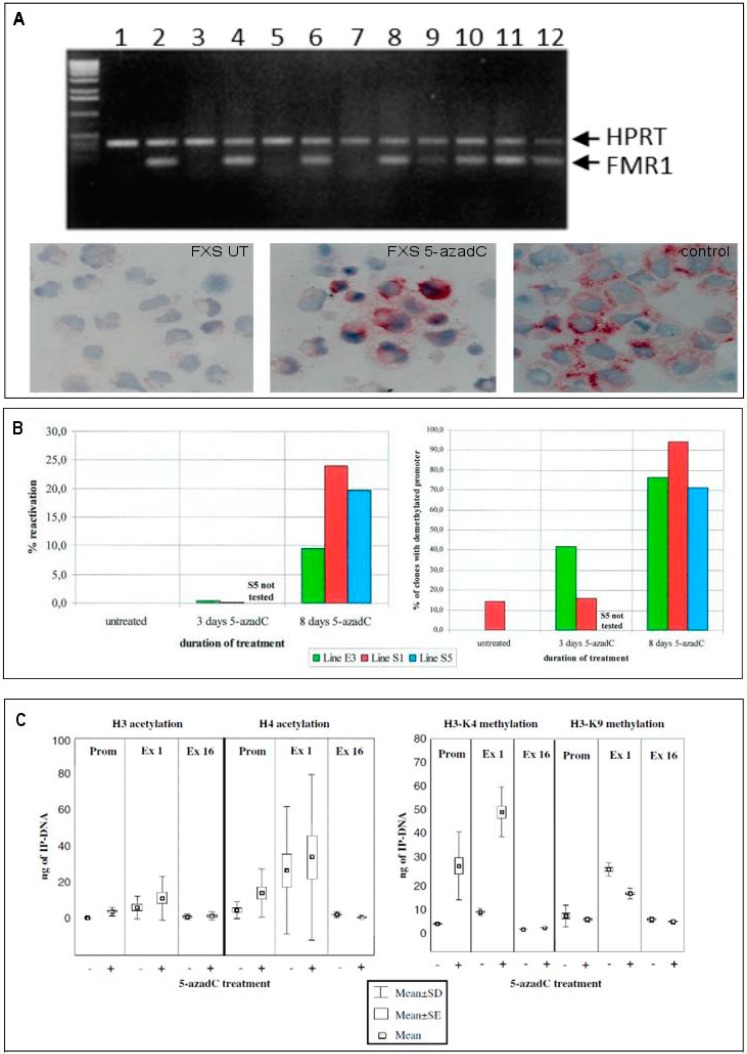
Epigenetic changes induced by 5-azadeoxycytidine (5-azadC) treatment. The use of 5-azadC on different FXS lymphoblasts produce *FMR1* transcript reactivation as shown by RT-PCR, and a partial rescue of translation demonstrated by immunocytochemistry (Panel **A**). Reactivation was quantified by real time PCR and the percentage of demethylated clones was evaluated by bisulphite sequencing of the CpG island of the *FMR1* promoter region (Panel **B**). The effects of 5-azadC on histone marks (acetylation of H3 and H4, increased methylation of H3K4) are reported in panel **C**. Data derived from Refs. [42,65,66].

**Figure 4:**
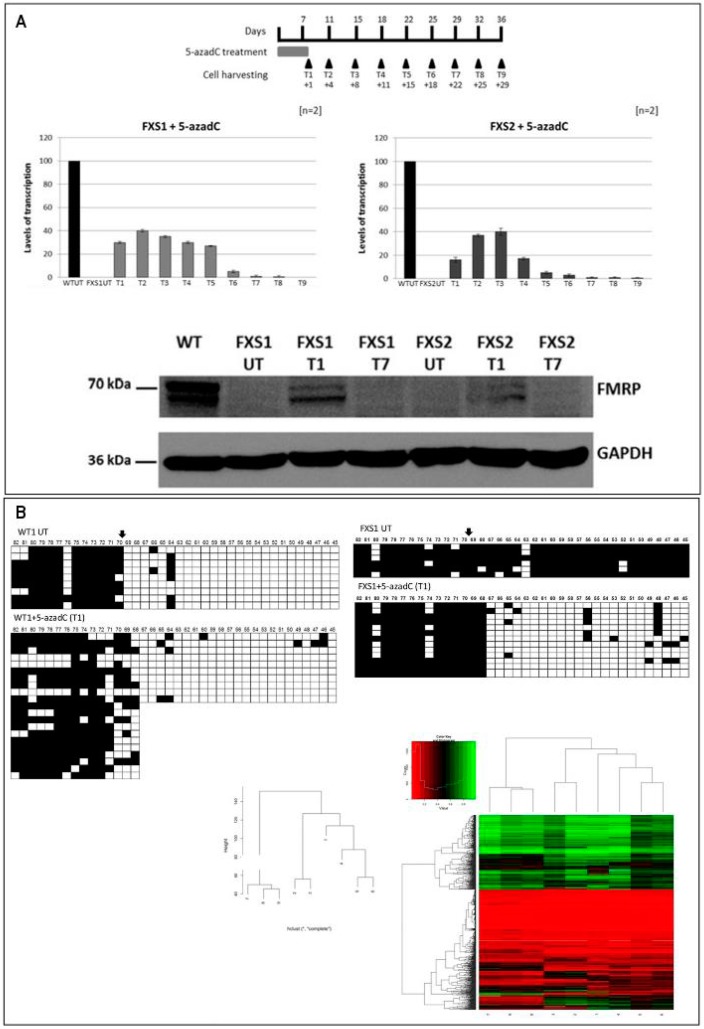
Long-lasting effect of 5-azadC and methylome analysis after treatment. In **A** are reported the transcription and translation results of a recent study [66]. The upper panel reports the scheme of 5-azadC treatment with the relative time points. Relative quantification of *FMR1*-mRNA by RT-PCR after treatments with 5-azadC of 2 different FXS lymphoblastoid cell lines showed increased *FMR1*-mRNA expression at T3 (8 days after last drug administration), decreasing at T6 (18 days after last drug administration) and T7 (22 days after last drug administration). Western blot with antibody against FMRP and GAPDH on FXS cell extracts demonstrated that after treatment the expression of FMRP was restored and disappeared after 22 days (T7) from the end of the treatment (Panel **A**). Panel **B**. Bisulphite sequencing of the methylation boundary including the CpG island of the *FMR1* promoter region before (top-left) and after (bottom-left) treatment with 5-azadC of WT cells showed no substantial modification of the methylation profile, while an almost complete demethylation of the promoter region, not affecting the methylation boundary was observed in FXS cells (top right). The methylation boundary is indicated by the arrow. Dendrogram of the methylation profile of the analyzed samples demonstrated a clustering of untreated samples compare to treated ones. The heat map shows some changes in the methylation profile after treatment, that however do not reach statistical significance (*p* > 0.05). Data derived from ref. [67].

DNA methylation likely represents the main epigenetic mark that switches off the expanded gene. DNA demethylation can be obtained with 5-azacytidine (5-azaC) or, more efficiently, with 5-azadC, that is incorporated as analog of deoxycytidine during cell replication and irreversibly blocks DNA methyltransferases [64]. Several studies have been performed to try to restore the activity of *FMR1* gene in vitro by inducing DNA demethylation with 5-azadC in lymphoblastoid cell lines from FXS patients. In 1998, we first achieved in vitro reactivation of the *FMR1* FM by treating FXS lymphoblastoid cell lines with 5-azadC for 7 consecutive days [65]. The reactivation was concomitant with partial DNA demethylation and partial restoration of FMRP production providing evidence that hypermethylation of the *FMR1* gene and not amplification of the CGG repeat is the major determinant in abolishing FMRP production [65]. Subsequent experiments refined the understanding of the reactivation process by analyzing the methylation status of individual CpG sites in the *FMR1* promoter region before and after 5-azadC treatment through bisulphite sequencing analysis. We demonstrated that 5-azadC-induced demethylation is partial and transient. After 4 weeks from 5-azadC withdrawal, the *FMR1* promoter resumed its methylated status [66]. To better understand the mechanisms of the *FMR1* gene reactivation, we undertook a systematic study of its epigenetic status, testing the acetylation and methylation of histones H3 and H4, in three different regions of the gene; promoter, exon 1 and exon 16 before and after treatment of FXS lymphoblastoid cell lines with 5-azadC for 7 consecutive days. The treatment induced histone acetylation, as well as methylation of H3K4, while only partly reducing H3K9 methylation [42]. These epigenetic changes appeared to restore an euchromatic configuration of the *FMR1* promoter effectively transforming a MFM (inactive) into an UFM (active) (Figure 3). In a more recent study we also demonstrated that the demethylating effect of 5-azadC on genomic DNA is not random, but rather restricted to the promoter region of *FMR1*, while the methylation boundary was not affected by treatment. Furthermore, the reactivating effect of 5-azadC was shown to last longer than previously thought (10–15 days after the last dose of the drug) [67]. In Figure 4 are reported the main results of this latter paper. Along the same line, Bar-Nur et al. [68] treated FXS-iPS cells and their derived neurons with 5-azaC and observed a significant *FMR1* reactivation after treatment. 

A limitation to the possible clinical use of 5-azadC is represented by its toxicity. While 5-azaC and 5-azadC are generally well tolerated in the treatment of hematological malignancies [69], the effects of a long-term treatment are unknown. A second obstacle is the apparent requirement for cell division in order for 5-azadC to be effective, even though two reports suggest that 5-azadC may require minimal or no incorporation into DNA to effectively reduce levels of DNA methyltransferase DNMT1 [70,71]. Nonetheless, there are good reasons for trying to identify mutant *FMR1* reactivating compounds having limited or no toxicity, such as, for instance, histone acetylating drugs. We showed that histone deacetylase inhibitors (butyrate and phenylbutyrate) alone did not reactivate *FMR1* in FXS lymphoblastoid cell lines, but were synergistic with 5-azadC in reactivating the silent gene [72].

**Figure 5:**
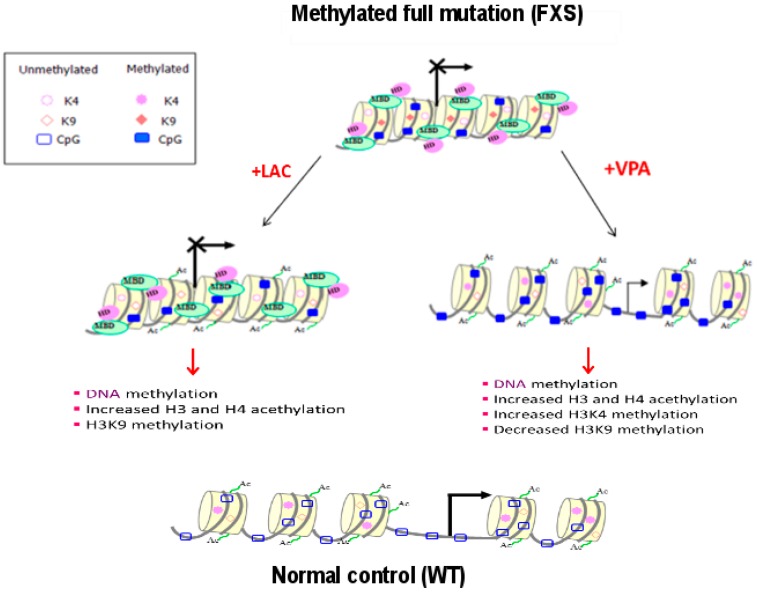
Major epigenetic modifications at the *FMR1* locus after histone acetylating treatments. In anormally active WT allele a permissive euchromatic configuration is present (bottom), while in methylated full mutation (FXS) the heterochromatic configuration does not allow transcription (top). The use of L-acetylcarnitine (LAC) on FXS induces an increase of H3 and H4 acetylation without DNA demethylation and transcriptional reactivation (middle left). Valproic acid (VPA) treatment shows a slight transcriptional activity with hyperacetylation of H3 and H4 and methylation of H3K4, while H3K9 methylation remains unmodified. MBD, methyl-binding domain protein; HD, histone deacethylases; Ac, histone acetyl groups.

Valproic acid (VPA), which acts as histone deacetylases inhibitor but not as DNA demethylator, was shown to have a modest effect as transcriptional reactivator of mutant *FMR1* in vitro [73]. VPA is widely used to treat epilepsy and bipolar disorder and is also a potent teratogen, it activates Wnt-dependent gene expression, similar to lithium, the mainstay of therapy for bipolar disorder [74]. In a preliminary safety clinical trial, 10 FXS subjects were treated with VPA for 6 months, showing a decrease in the hyperactivity phenotype [75]. Similar findings had been previously obtained in a clinical trial with L-acetylcarnitine (LAC) [76], a natural compound that can efficiently increase histone acetylation, but is not sufficient to cause *FMR1* reactivation when used alone in vitro [41]. Figure 5 reports the major epigenetic modifications observed after VPA and LAC treatment in vitro.

Besides drugs to treat FXS, further perspectives may be taken into account, based on the newly available CRISPR/Cas9 gene editing technique. Recently a targeted deletion of trinucleotide repeats restoring *FMR1* gene expression was produced in FXS-ESC and iPS derived-neuronal cells. These results provide further insights into the molecular mechanisms of FXS and towards future therapies of trinucleotide repeat disorders [77].

## 3. Conclusions

Among rare genetic disorders, FXS appears to be more amenable than others to an effective pharmacological treatment. FXS is strictly monogenic, practically all patients having the same mutation; the mutation does not affect the coding sequence of the gene but rather its reversible epigenetic status; the pathogenic mechanism is relatively well elucidated; the phenotype (ID and behavioral problems) ranges from mild to moderate, and it does not normally include structural defects of tissues or organs. However, as testified by several clinical trials, the effective correction of a genetic defect continues to be a tremendous challenge, still requiring a wider basic knowledge of the pathophysiology underlying the disease.

## Figures and Tables

**Table 1:** Major epigenetic modifications at the *FMR1* locus in transcriptionally active (wild type (WT) and unmethylated full mutation (UFM)) and inactive fragile X syndrome (FXS) alleles.

	WT	FXS	UFM
DNA methylation	− (absent)	+ (present)	− (absent)
H3 and H4 acetylation	+	−	+
H3K4 methylation	+	−	+
H3K9 methylation	−	+	+/−
H3K27 dimethylation	+	−	+
H3K27 trimethylation	−	+	−
